# The Acoela: on their kind and kinships, especially with nemertodermatids and xenoturbellids (Bilateria incertae sedis)

**DOI:** 10.1007/s13127-012-0112-4

**Published:** 2012-09-29

**Authors:** Johannes G. Achatz, Marta Chiodin, Willi Salvenmoser, Seth Tyler, Pedro Martinez

**Affiliations:** 1 Department of Genetics, University of Barcelona, Av. Diagonal, edifici annex, planta 2a, 08028 Barcelona, Spain; 2 Department of Evolutionary Developmental Biology, University of Innsbruck, Technikerstrasse 25, 6020 Innsbruck, Austria; 3 School of Biology and Ecology, University of Maine, 5751 Murray Hall, Orono, ME 04469 USA; 4 Institució Catalana de Recerca i Estudis Avançats (ICREA), Passeig Lluís Companys, 23, 08010 Barcelona, Spain

**Keywords:** Acoelomorpha, *Xenoturbella*, Morphology, Development, Systematics, Phylogeny

## Abstract

Acoels are among the simplest worms and therefore have often been pivotal in discussions of the origin of the Bilateria. Initially thought primitive because of their “planula-like” morphology, including their lumenless digestive system, they were subsequently dismissed by many morphologists as a specialized clade of the Platyhelminthes. However, since molecular phylogenies placed them outside the Platyhelminthes and outside all other phyla at the base of the Bilateria, they became the focus of renewed debate and research. We review what is currently known of acoels, including information regarding their morphology, development, systematics, and phylogenetic relationships, and put some of these topics in a historical perspective to show how the application of new methods contributed to the progress in understanding these animals. Taking all available data into consideration, clear-cut conclusions cannot be made; however, in our view it becomes successively clearer that acoelomorphs are a “basal” but “divergent” branch of the Bilateria.

## Introduction

**Fig. 1:**
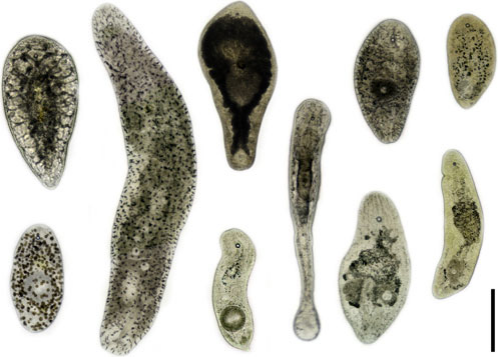
Images of various live acoels found in a beaker of sublittoral sand from the Indian Ocean. Animals are oriented with the anterior end to the top. Note the statocyst in all and mature oocytes in some animals. Scale bar: 200 μm

**Fig. 2:**
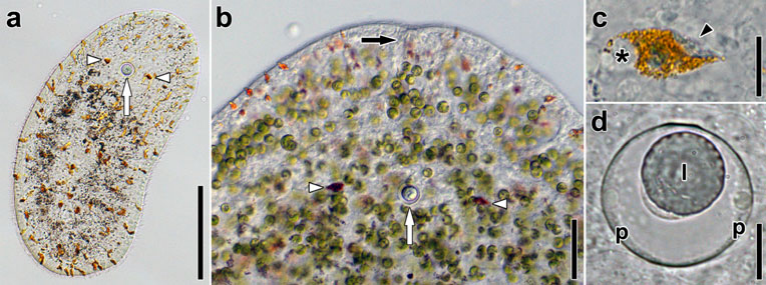
Images of sensory structures of live *Symsagittifera roscoffensis*. **a** Hatchling. *Arrowheads* point to eyes, *arrow* to statocyst. Note absence of symbionts and presence of orange rhabdoids. **b** Anterior end of adult with symbionts and rhabdoids. *White arrowheads* point to eyes, *white arrow* to statocyst, *black arrow* to frontal organ. **c** Eye of an adult. *Asterisk* marks nucleus, *arrowhead* points to concrements. **d** Statocyst of an adult. Abbreviations: *l* lithocyte; *p* parietal cells. Scale bars: **a** 100 μm; **b** 50 μm; **c** 10 μm; **d** 10 μm

**Fig. 3:**
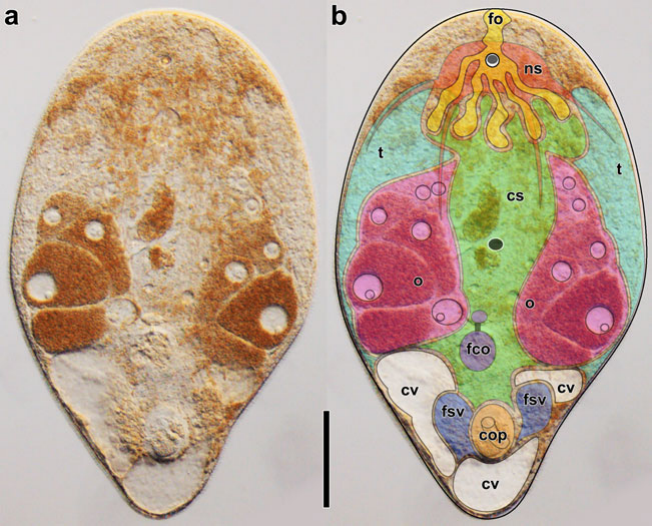
Image of a mature and live specimen of *Isodiametra pulchra* without (*left*) and with superimposed colors (*right*) to illustrate the general morphology of acoels. From top to bottom: *yellow*: frontal organ (*fo*); *red*: nervous system (*ns*); *green*: central syncytium (*cs*); *cyan*: testes (*t*); *pink*: ovaries (*o*); *gray*: mouth; *purple*: female copulatory organs (*fco*) composed of seminal bursa, bursal nozzle, and vestibulum (from posterior to anterior); *white*: chordoid vacuoles (*cv*); *blue*: false seminal vesicles and prostatoid glands (*fsv*); *orange*: male copulatory organ (*cop*) composed of muscular seminal vesicle and invaginated penis. Scale bar: 100 μm

Acoels are bilaterally symmetric, microscopic worms, typically in the millimeter-size range, that are found predominantly in benthic marine habitats. They can easily be recognized by the presence of a characteristic statocyst at the anterior end (see sensory organs; Figs. 1, 2a, b, d). Most are translucent or somewhat milky, but some are colored by pigmentation, by algal symbionts, or by glandular secretions called rhabdoids (Figs. 1, 2a, b, 5a). Their body shapes correlate with their habitat: species living in sand are long and slender, those moving on or in mud are compact and droplet-shaped, those moving on or beneath stones and corals are broad and flat, epiphytic species have ventrally enrolled sides, and pelagic species have a disc-shaped body or enrolled sides (Figs. 1, 3, 5a).

Acoels are acoelomate, the space between gut and body wall being filled with parenchymal cells that occasionally contain chordoid vacuoles and the insunk bodies of epidermal and gland cells. The name ‘acoel’ comes from their lack of a cavity in the gut, which is typically a solid syncytium.

Of the nearly 400 described species (Tyler et al. 2012, The Turbellarian Taxonomic Database, http://turbellaria.umaine.edu; Wallberg 2012, The Stylet–Diversity and Systematics of Acoela and Nemertodermatida, http://acoela.myspecies.info), by far the majority are free-living, but seven are parasites or endosymbionts in the digestive system of echinoderms (Jennings 1971), and two are found in fresh water (Ax and Dörjes 1966; Faubel and Kolasa 1978). Their diet varies as much as their habitat, ranging from bacteria and unicellular algae to crustaceans, small bivalves, and worms (including other acoels); some are known for cannibalism (e.g., *Conaperta flavibacillum*).

## Morphology

### Epidermis

Like most microscopic worms, acoels move predominantly by ciliary gliding. The epidermis is multiciliated, and the cilia have the common configuration of nine peripheral microtubule doublets and two central microtubules (9 × 2 + 2). The shape of the cilia is distinctive, having a marked shelf at the tip where the doublets 4–7 terminate (Tyler 1979; Ehlers 1985; Smith and Tyler 1985a; Smith et al. 1986; Rieger et al. 1991). Even more distinctive of the cilia is their rootlet system, which interconnects them: from the major, rostrally directed rootlet on each cilium, two lateral rootlets project and connect to the tips of the adjacent cilia, and from a caudal rootlet two bundles of fibers project to join the knee-like bend of those same adjacent rootlets (Hendelberg and Hedlund 1974; see Fig. 1 F in Rieger et al. 1991).

### Glands

**Fig. 4:**
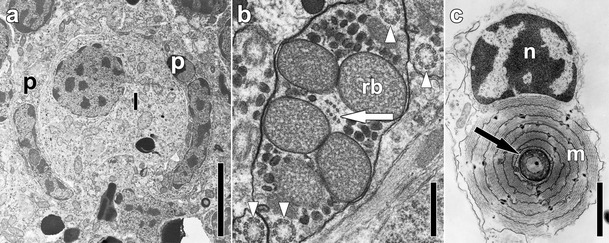
Electron micrographs of structures with phylogenetic significance. **a** Statocyst of a hatchling of *Isodiametra pulchra* with two parietal cells (*p*) and a lithocyte (*l*). **b** Sperm of *Convoluta niphoni* (Convolutidae) with axial microtubules (*white arrow*) and axonemes without central microtubules (*white arrowheads*). **c** Extrusion apparatus of *Convolutriloba hastifera* consisting of a sagittocyst (*black arrowhead*) and a wrapping muscle mantle. Abbreviations: *m* muscle mantle; *n* nucleus of muscle mantle; *rb* refractive body; *p* parietal cell. Scale bars: **a** 4 μm; **b** 0.5 μm; **c** 2 μm

Unicellular glands that typically richly populate the epidermis include the above-mentioned rhabdoid glands (Smith et al. 1982), which may be colored, and mucous glands. Glands occurring at special positions include sagittocytes that produce needle-shaped extrusomes (sagittocysts, Fig. 4c) predominantly near the reproductive organs; prominent mucous glands of the frontal organ that discharge together through a pore at the anterior terminal end of the body (Smith and Tyler 1985b, 1986; Klauser et al. 1986; Smith et al. 1986; Rieger et al. 1991; Figs. 2b, 3); and frontal glands of a variety of types that discharge near the anterior tip. The nuclei of all these gland cells with the exception of most pigment cells are usually positioned below the body-wall musculature.

### Sensory organs

**Fig. 5:**
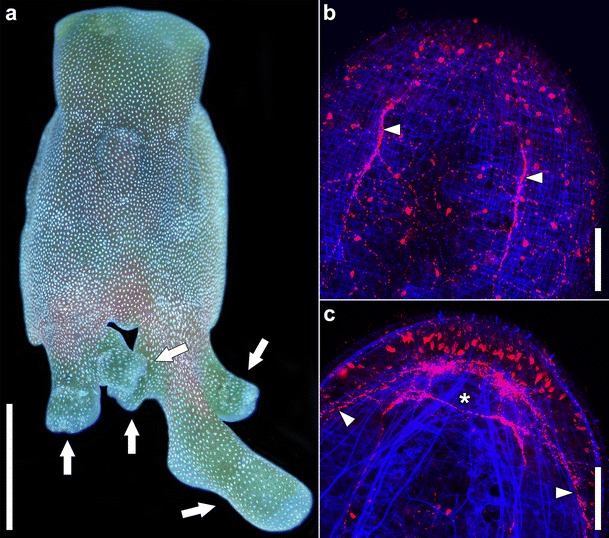
**a** Image of a live specimen of *Convolutriloba retrogemma* reproducing asexually by budding. *White arrowheads* point to buds. Note the reversed polarity. **b**, **c** CLSM projections showing muscles (*blue*) and serotonin-like immunoreactive nervous system (*red*) in dorsal (**b**) and central (**c**) planes of a mature *Isodiametra pulchra*. *White arrowheads* point to neurite bundles, *asterisk* marks the position of the statocyst. Scale bars: **a** 1 mm; **b** and **c** 50 μm

Specifically distinctive of acoels, the statocyst comprises a lithocyte bearing one statolith encompassed in a capsule formed by two lining parietal cells (Ehlers 1985; Figs. 2d, 3, 4a). Occasionally, animals that have been reproduced asexually may lack the statocyst (Hanson 1960, Hendelberg and Åkesson 1988; Åkesson et al. 2001; see Fig. 5a), whereas panther worms (Hofsteniidae) have been reported to occasionally possess more statoliths after regeneration of the anterior body region (Steinböck 1966).

In a small percentage of species paired eyespots, which are probably photoreceptive, occur at the anterior end (Figs. 2a, b, c). Lanfranchi (1990) described the eyespots of *Otocelis rubropunctata* as specialized epidermal cells with typical 9 × 2 + 2 cilia and with pigment granules and many synapses and axonemal outgrowths on the basal surface, but he was unable to prove photoreceptive function. Yamasu (1991) suggested the photoreceptive capacity of the eyes of *Praesagittifera naikaiensis* by relating experimental ablation with behavioral assays. In this and some other species, the eyespots don’t have ciliary or rhabdomeric elements but consist of a pigment cell containing a vacuole with refractive inclusions called concrements and up to three nerve cells to relay the stimulus. The same configuration of cells has also long been known in *Convoluta convoluta* (Popova and Mamkaev 1985), and such eyespots have subsequently been recognized to be characteristic for a derived group within the Acoela, the Convolutida (Hooge and Tyler 2005; Achatz et al. 2010). In all likelihood, many species of the Acoela can detect light (and behave accordingly) through photoreceptive sensory cells of the epidermis—cells that are difficult to identify because they are not accompanied by pigment cells.

Other known sensory organs in acoels are single-celled receptors, which are mostly monociliary. These can be classified into several types on a morphological basis (Todt and Tyler 2007 and references therein), each type occurring in a specific region of the body that is species-specific (Todt and Tyler 2007; Bery et al. 2010).

### Nervous system

The nervous system itself consists of a supramuscular plexus, a submuscular plexus, 3–5 pairs of longitudinal neurite bundles (terminology after Richter et al. 2010), and a brain, which is shaped like a ring, a barrel, or a bilobed mass, with a complex connectivity of various fibers forming connectives and commissures (Raikova et al. 1998; Reuter et al. 2001a, b; Gaerber et al. 2007; Achatz et al. 2010; Bery et al. 2010; Semmler et al. 2010; Figs. 3, 5b, c). Serotonin-like immunoreactive (Raikova et al. 1998, 2004a; Reuter et al. 2001a, b; Gaerber et al. 2007; Semmler et al. 2010; Figs. 5b, c), RFamide-like immunoreactive (Raikova et al. 2004a; Reuter et al. 1998), and cholinergic (Gaerber et al. 2007; Bery and Martinez 2011 and references therein) parts of the nervous system have been revealed by immunohistochemistry and conventional histochemistry. The neurite bundles are generally distributed evenly around the anteroposterior axis and are similar in diameter; however, the dorsal or ventrolateral neurite bundles may be more pronounced (Rieger et al. 1991; Raikova et al. 1998, 2001).

### Muscles

Besides ciliary gliding, acoels use muscles to move. Abundant dorsoventral muscles serve to flatten the body, and the musculature of the body wall and parenchymal muscles generate bending, shortening, and lengthening movements. The body-wall musculature comprises circular-, diagonal-, longitudinal-, longitudinal crossover-, spiral-, U-shaped-, reversed U-shaped, and pore muscles (Hooge 2001; Tekle et al. 2005; Semmler et al. 2008; Achatz et al. 2010; Figs. 5b, c). The arrangement and complexity of the ventral body-wall musculature led Tyler and Rieger (1999) to hypothesize that it serves in ingesting food and so functionally makes up for the lack of a true pharynx.

### Pharynx

Pharynges are present in the acoel families Diopisthoporidae, Hallangidae, Hofsteniidae, and Solenofilomorphidae, and the genera *Oligochoerus* (Convolutidae) and *Proporus* (Proporidae). Detailed morphological analyses of these pharynges show that they are very diverse with respect to musculature, the nature of the lining cells, and the types of receptors present (Karling 1974; Crezée 1975; Doe 1981; Rieger et al. 1991; Todt and Tyler 2007; Todt 2009). Nowhere else in the animal kingdom is the position of the mouth as variable as it is in acoels. Even though it is most commonly situated mid-ventrally, the mouth can be anywhere from subterminally at the anterior (*Proporus*, *Hallangia*, *Hofstenia,* and some species in the Isodiametridae) to terminally on the posterior end (*Diopisthoporus*) and anywhere in between along the ventral midline.

### Gut

The gut is syncytial and lacks a lumen in most investigated species and is therefore commonly termed a central syncytium, but central parenchyma is a common term as well (Fig. 3); however some species, notably representatives of the Paratomellidae, have a lumen without an epithelial lining (its cells are parenchymal, packed in a jumble, and lack the aligned polarity and cell junctions characteristic of epithelia—Smith and Tyler 1985a; Ehlers 1992a). All acoels hitherto studied, covering a wide range of sizes and phylogenetic distribution (compare species studied in Smith and Tyler 1985a and phylogeny of Jondelius et al. 2011), lack glandular cells, as would be typical of the gut of most animals (including the sister group, the Nemertodermatida—see below) in the digestive tissue.

### Excretory organs

No typical excretory organs have been found in acoels. Cells that resemble the cyrtocytes of protonephridia (so-called “pulsatile bodies” with waving cilia found below the epidermis) have been shown to be degenerating epidermal cells that are in the process of being resorbed (Mamkaev 1967; Tyler et al. 1989; Ehlers 1992b; Lundin 2001). Cells lacking cilia and resembling the canal cells of protonephridia (with a branching system of lacunae and tubules connecting to the outside) have been proposed to be excretory cells in *Paratomella rubra* (Ehlers 1992c).

### Symbionts

Symbiotic algae are found in many acoels living in sun-exposed habitats (Figs. 2b, 5a) and are essential for the survival of the host (Shannon and Achatz 2007). These can be either zoochlorellae or zooxanthellae, or both together in some species (see Achatz et al. 2010 for more detail). Transfer is commonly horizontal, meaning that the symbionts are acquired anew by each generation. Vertical transmission, whereby the symbionts are passed to the next generation in the egg, is known for *Amphiscolops carvalhoi* (Marcus 1952) and *Waminoa brickneri* (Barneah et al. 2007). The establishment of symbioses with algae happened at least twice within the Acoela (Achatz et al. 2010).

### Gonads

Acoels are simultaneous or slightly protandric hermaphrodites. The gonads are always asaccate (asacular in Rieger et al. 1991), meaning that the germ cells are not lined and separated from the surrounding parenchyma by specialized tissue called tunica (Fig. 3; for exceptions see also below—What is primitive in the Acoelomorpha?; Rieger et al. 1991, pp 88 and 93; the notion of Boone et al. 2011 that testes in acoels can be saccate must be a misinterpretation of the literature). The position of the ovaries and testes is highly variable even with regard to each other; they can be paired or unpaired, and in a few species (e.g., *Antigonaria*) their germinative zone is mixed, producing both sperm and ova (Rieger et al. 1991). The oocytes are entolecithal and in many cases accompanied by accessory cells, but contrary to occasional claims (Mark 1892; Dörjes 1968; Winsor 1988), the ovary is never differentiated into germarium and vitellarium (Achatz et al. 2010). Sperm are described in more depth as they provide important characters for the internal phylogeny of acoels. During the early development of sperm—spermatogenesis—spermatids grow two free flagella at the distal end, which are subsequently incorporated into the body of the sperm in a proximal direction. They run its entire length or close to the distal end of the nucleus, which is positioned at the proximal end of the sperm (Hendelberg 1969, 1977). The flagella lose their membrane after fusion, but the axonemes remain. In most cases these axonemes show the typical configuration of nine peripheral microtubule doublets and two central microtubules (as in locomotory cilia); however, in some species there is only one central microtubule (9 × 2 + 1) or none (9 × 2 + 0—see Fig. 4B). There are additional microtubules in the cytoplasm of the sperm, most likely to provide some rigidity to the cell. These cytoplasmic microtubules are positioned either under the plasma membrane, forming a kind of cytoskeletal sheath (so-called cortical microtubules) or run through the central axis of the sperm in between the two axonemes (axial microtubules) (Figs. 4b, 7).

### Canal system

Sperm usually aggregate within spaces in the parenchyma close to the male copulatory organ. If these spaces are encompassed by specialized tissue (including muscles that provide pressure to eject the sperm and secretions), they are called seminal vesicles; if the parenchyma has no obvious differentiation, they are called false seminal vesicles; however, both types can be present in the same individual (Fig. 3). The male copulatory organs are highly diverse and range in general anatomy from being absent or simple invaginations of the body wall (antrum) to complicated arrangements comprising muscular or sclerotized parts that are combined with glandular parts and muscular bulbs that provide pressure for the ejection of sperm (Westblad 1948; Dörjes 1968). The male gonopore can be situated anteroventrally along the ventral midline up to the posterior end, its position, as well as that of the copulatory organ, depending on the position of the testes and the direction of maturation of the sperm.

**Fig. 6:**
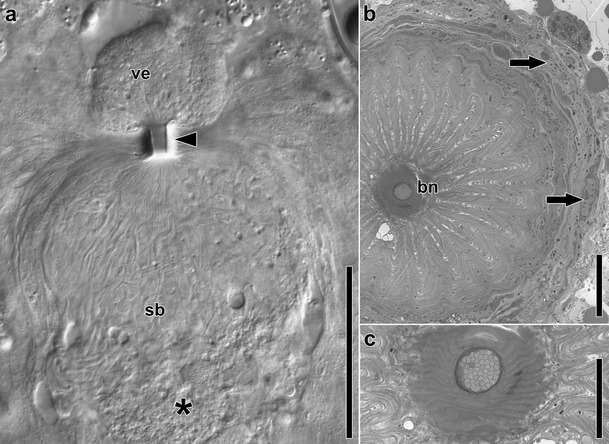
Female copulatory organs in *Isodiametra pulchra*. **a** Image of female copulatory organs in a live and squeezed specimen. Note the mass of elongated and convoluted sperm in the seminal bursa (*sb*) that merge towards the bursal nozzle (*arrowhead*) and a few “heads” extending into the vestibulum (*ve*). *Asterisk* marks bursal stalk connecting the bursa with the digestive parenchyma, *arrowhead* points to bursal nozzle. **b** Electron micrograph showing cross section through the bursal nozzle (*bn*). *Arrows* point to nuclei of cells of the bursal wall. **c** Counterclockwise rotated detail of **b**. Note the density of sperm in the duct of the bursal nozzle. Abbreviations: *bn* bursal nozzle; *sb* seminal bursa; *ve* vestibulum. Scale bars: **a** 50 μm; **b** 10 μm; **c** 5 μm

The female copulatory organ consists of gonopore(s), vagina(e), seminal bursa(e), and one or many bursal nozzles (Figs. 3, 6a, b, c), but some or all of these parts can be missing, leaving the animal with a kind of inconspicuous bursal tissue or no obvious adaptation at all. A seminal bursa is a distinct “pocket” made up of parenchymal cells that serves to store and digest sperm received from a mating partner (Brüggemann 1985a; Petrov et al. 2006; Achatz et al. 2010; Fig. 6a). Bursal nozzles are structures, stiffened by F-actin-rich cells, that accompany or are part of the seminal bursa; they appear to select and modify sperm (Brüggemann 1985a; Petrov et al. 2006; Achatz et al. 2010; Figs. 6a, b, c).

## Reproduction and development

### Sexual reproduction

Fertilization is always internal; the mode of copulation varies considerably and seems to be related to the environment (Apelt 1969). Among the modes of sperm transfer are mutual exchange (Hyman 1937; Costello and Costello 1938; Westblad 1946; Apelt 1969), hyperdermal transmission (Bush 1975), and hypodermal injection (Apelt 1969). In general, in the first two cases, a simple opening in the epidermis, an antrum, or a soft, muscular penis serves to transfer sperm; in the last case the epidermis of the partner is commonly punctured with sclerotized accessory structures like needles or a stylet.

Eggs are laid individually or in clusters through the mouth, the female gonopore, or through rupture of the body wall (Costello and Costello 1939; Apelt 1969; see Rieger et al. 1991).

### Development

Embryonic development is direct and follows a distinct spiral duet cleavage pattern that likely originated independently from the common quartet spiral cleavage of the lophotrochozoan phyla (Bresslau 1909; Apelt 1969; Boyer et al. 1996; Henry et al. 2000). The cleavage pattern is only known for a few species of acoels, all belonging to the Crucimusculata, with the exception of *Diopisthoporus*, which is viviparous and in which embryonic development is difficult to follow. Nevertheless, it is clear that cleavages are spiral and that the second, asymmetric and horizontal cleavage leads to the production of micromeres (Apelt 1969). As in quartet spiral cleavage, the first horizontal cleavage is unequal and so produces micromeres, but it occurs at the two-cell stage instead of the four-cell stage, so the micromeres appear as duets instead of quartets. The micromeres arise in a leiotropic direction with respect to the animal-vegetal axis, as do all subsequent micromeres, unlike the micromeres in spiral quartet cleavage, which are alternately leio- and dexiotropic. Also distinct from spiralian cleavage is its more bilateral nature: the sagittal plane (and so the antero-posterior axis) of the adult is defined by the first cleavage, whereas this plane and axis lie oblique to the quadrants in quartet spiral cleavage (Henry et al. 2000). The first, second, and third micromere duets give rise to all ectodermal structures, while endodermal (parenchyma) and mesodermal (muscles) structures are derived from the third duet of macromeres. Gastrulation occurs by growth of the micromeres upon the macromeres, and the mouth is formed at a site other than the blastopore (Boyer et al. 1996; Henry et al. 2000).

Unlike the canonical spiralian development, acoel duet spiral development shows no ecto-mesoderm source. Internal tissues arise either by delamination—that is, mitoses are oriented so as to produce digestive parenchyma, musculature, and nervous tissue toward the interior of the embryo (of *Neochildia fusca*: Ramachandra et al. 2002) or by immigration of cells that form the endoderm and mesoderm (in *Convolutriloba longifissura*: Hejnol and Martindale 2008a). By the time gastrulation is complete, the embryo looks layered: the outermost layer is the epidermal primordium, a middle layer contains progenitors of muscles and neurons, and the innermost cells are those that will develop into the digestive syncytium. The segregation of organs starts afterwards, when the ciliated epithelium plus sub-epithelial muscle fibers form and when the nervous system begins to differentiate at the anterior end of the embryo.

While knowledge of the development of the nervous system remains incomplete, the development of the musculature of the body wall has been studied in two species, *Isodiametra pulchra* and *Symsagittifera roscoffensis*. By means of labeling of F-actin filaments, Ladurner and Rieger (2000) and Semmler et al. (2008) found that primary myocytes appear in the anterior half of the embryo of both species about halfway through development. Complete circular fibers form before longitudinal fibers, in an anteroposterior progression. In *I. pulchra* the first myocytes appear as single cells separated from each other in latitudinal positions; by elongating and connecting to each other with fine endings, these fibers completely encircle the embryo (Ladurner and Rieger 2000). Longitudinal fibers appear in a bilateral pattern and follow a similar developmental course. In contrast, in *S. roscoffensis*, the circular, longitudinal and diagonal primary myocytes seem to form simultaneously (Semmler et al. 2008). In both species, the primary muscle fibers serve as a template for the formation of secondary and further muscle fibers, a mechanism that is also used during muscle regeneration (see below). Accessory muscles, such as the sphincter muscles of the mouth, develop shortly before hatching.

### Asexual reproduction

While all acoels reproduce by sexual reproduction, many can also reproduce asexually through a variety of mechanisms. Paratomy—the preformation of organs before separation—occurs in the Paratomellidae (Dörjes 1966) and results in a chain of zooids; architomy, by which the organs form after the separation of mother and daughter, is common in the family Convolutidae, namely among the genera *Adenopea* (du Bois-Reymond Marcus 1955), *Amphiscolops* (Hanson 1960), and *Symsagittifera* (Marcus and Macnae 1954), and in species of *Convolutriloba* (Bartolomaeus and Balzer 1997); and budding occurs in other species of *Convolutriloba*, whereby the daughter individual develops with its anteroposterior axis perpendicular to or reversed in relation to that of the mother (Hendelberg and Åkesson 1988; Åkesson et al. 2001; Shannon and Achatz 2007; Sikes and Bely 2008, 2010; see Fig. 5a).

### Regeneration

Acoels exhibit great regenerative capacity after fission or after experimental amputation (see Egger et al. 2007). In all species studied to date, the process involves an initial muscle contraction that helps to close the wound. Muscle fibers that develop in the wound area are largely randomly oriented initially and only gradually achieve their orthogonal arrangement. Pre-existing muscle fibers and longitudinal neurite bundles invade the newly formed blastema and serve as a template for the differentiation of new myocytes and neurons (Gschwentner et al. 2001; Gaerber et al. 2007; Sikes and Bely 2008; Bery and Martinez 2011; Chiodin et al. 2011). Development, regenerative processes, and tissue homeostasis are controlled by somatic stem cells called neoblasts (De Mulder et al. 2009). These neoblasts usually show a high nucleus/cytoplasm ratio with little cytoplasmic differentiation and are referred to as totipotent, meaning that they can differentiate into all cell types. Somatic neoblasts are localized exclusively within the parenchyma, in contrast to the epidermal positions of stem cells in other metazoans, with the exception of rhabditophoran flatworms (for more detail, see De Mulder et al. 2009 and Egger et al. 2009). The germ cells and a subpopulation of somatic neoblasts in *I. pulchra* express a homolog of the gene *piwi*, the silencing of which does not affect cell proliferation in adult worms but does affect their ability to produce offspring; silencing also eventually kills juveniles treated during development (De Mulder et al. 2009). In most bilateral animals, *piwi* is a germline marker (and is found in the germline of *I. pulchra* as well), whereas it is found only in somatic stem cells of sponges, cnidarians, and rhabditophoran flatworms; thus its function in stem-cell specification must be primal (De Mulder et al. 2009).

## Phylogenetic relationships within the Acoela

**Fig. 7:**
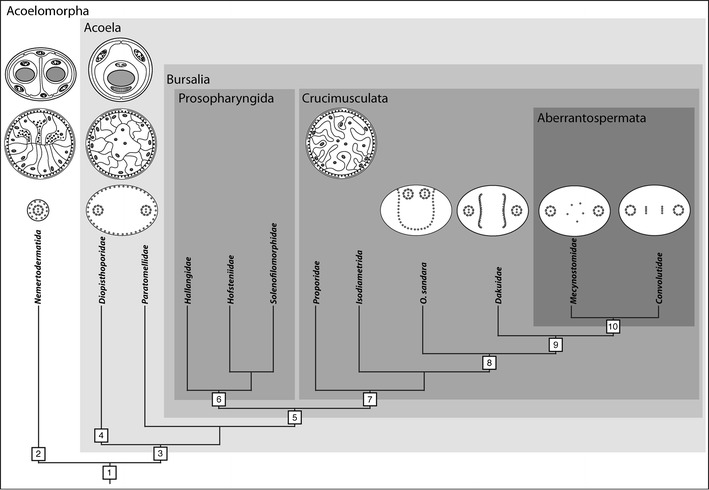
Cladogram of the Acoelomorpha with partial family-level systematics of the Acoela. *1*. Multiciliated epidermis, ciliary rootlet system, frontal organ, basiepidermal nervous system with ring-shaped brain. *2*. Statocyst with two lithocytes (statoliths) and many parietal cells, sperm with cork screw-like morphology. *3*. Statocyst with one lithocyte (statolith) and two parietal cells, brain sunk below body wall, lateral fibers at knee of rostral rootlet, biflagellated sperm; digestive system becomes depolarized. *4*. Position of mouth at the posterior end. *5*. Specialized parenchymal tissue for reception, storage, and digestion of sperm (seminal bursa). *6*. Subterminal pharynx at anterior end. *7*. Ventral crossover muscles and highly branched wrapping cells. *8*. Cytoplasmic microtubules of sperm partially lose contact with membrane and change position toward the center of the cell. *9*. Cytoplasmic microtubules of sperm change position toward the center of the cell, stacked bursal nozzles with matrix and gland cells. *10*. Central microtubules in axonemes of sperm reduced to allow movement in more than one plane. General scheme after Achatz et al. (2010); schemes of cross sections through statocysts from Ehlers (1985), through bodies after Rieger and Ladurner (2003); systematics and branching after Jondelius et al. (2011)

As acoels only show a paucity of variable organs, and only rarely bear consistently sclerotized structures, they offer few characters on which to base classification. Additionally, their microscopic size makes them difficult to investigate. The first acoel described, *Convoluta convoluta*, was classified as a planaria simply by its overall similarity to better-known triclad turbellarians (Abildgaard 1806), and subsequent descriptions of acoel species variously reported acoels to have no nervous system (Uljanin 1870; Graff 1882) and confused the terminal pore of the frontal organ with the mouth opening (Graff 1891). Growing knowledge of acoel diversity (Graff 1905; Luther 1912; Westblad 1940, 1942, 1945, 1946, 1948; Marcus 1947, 1948, 1949, 1950, 1951, 1952, 1954) finally led to the construction of a stable family-level system by Dörjes (1968) that was based primarily on light microscopic traits of the male copulatory organ. However, Dörjes did not develop a phylogenetic hypothesis for the Acoela because, with the characters at hand, there was no striking transformation series between families. It was the progress in investigative tools that paved the way to clearer concepts of relationships. Electron microscopy made it possible to see details down to cellular substructures and provided more characters on which to establish similarities and differences, and by means of confocal laser-scanning microscopy, in combination with immunocytochemistry and fluorophore-tagged phalloidin (Figs. 5b, c), parts of the nervous system (Raikova et al. 1998, 2004a), the muscles of the body wall (Hooge 2001; Tekle et al. 2005), and the ducts and musculature of copulatory organs (Hooge and Tyler 2005) could be revealed with ease. By applying these techniques, sperm ultrastructure (Hendelberg 1977; Raikova et al. 2001; see Figs. 4b, 7) and body-wall musculature (Hooge 2001) could be discerned and provided a basis for the first substantial hypotheses of family interrelationships. Hooge et al. (2002) and Jondelius et al. (2011) confirmed and further expanded our understanding of these relationships through molecular sequence studies.

The most recent and most data-rich hypothesis of relationships is that of Jondelius et al. (2011); it covers rDNA and COI sequences from about a third of all described species, only missing data from the Anthroposthiidae, and the monotypic Antigonariidae, Nadinidae, and Taurididae (see Fig. 7 for a simplified phylogenetic scheme). In summary, the analysis shows that the Diopisthoporidae is the most basal family of the Acoela, followed by the Paratomellidae and a clade Jondelius et al. (2011) call Prosopharyngida, comprising the Hallangidae, Hofsteniidae, and Solenofilomorphidae. The basal position of these families is consistent with earlier claims based on morphology, especially for the Paratomellidae (Smith and Tyler 1985a; Ehlers 1992a; Raikova et al. 1997, 2001) and the Hofsteniidae and Solenofilomorphidae, the relationship of which was implied by their possession of a specific type of receptor with an enlarged main rootlet and a smaller posterior rootlet (Todt and Tyler 2007). However, as mentioned by Jondelius et al. (2011), *Hallangia proporides* does not easily fit in the Prosopharyngida, showing characters that are reflective of isodiametrids.

The five “basal” families are clearly set apart from the “higher acoels,” or Crucimusculata (as named by Jondelius et al. 2011), which are identified by the possession of ventral crossover muscles (Jondelius et al. 2011) but also wrapping cells (Smith and Tyler 1985a; see Fig. 7). Because many families within the Crucimusculata were recovered as paraphyletic, Jondelius et al. (2011) synonymized several of them: the Haploposthiidae and Polycanthiidae with Proporidae, Childiidae with Mecynostomidae, and Anaperidae and Sagittiferidae with Convolutidae; they also transferred species of the Otocelididae with copulatory needles and the genus *Philactinoposthia* to the Dakuidae.

Jondelius et al. (2011) also reconstructed the ancestral state via simultaneous analysis of gene sequence data and 37 morphological characters under parsimony and Bayesian optimality criteria. Characters such as the presence or absence of a vagina and seminal vesicle were shown to be uninformative to the phylogenetic relationships, whereas those of the copulatory organs were quite significant at the family level and those of the body-wall musculature at deeper backbone nodes (except in the Mecynostomidae and Proporidae, for which the genitals were reconstructed with a slightly stronger signal than the muscles). By means of these analyses even the characteristics of the common ancestor to all acoels could be determined with some accuracy. However, the results should be taken with a pinch of salt as the character analysis (how morphological characters are selected, how states are defined, delimited, coded, and ordered; Wiens 2001), which is as crucial for the analysis of morphological characters as is the alignment for the analysis of a molecular data set (Pleijel 1995; Freudenstein 2005), lacks accuracy. The presence of a stylet, for instance, was reconstructed in all deep nodes under the model based on Bayesian character reconstructions, with BPPs ranging between 0.95 and 0.97 (see Table 4 in Jondelius et al. 2011), and therefore the presence of a stylet is considered part of the ground pattern in acoels (see Fig. 9 in Jondelius et al. 2011), having been lost repeatedly within the clade. Yet, stylets in the Mecynostomidae are composed of tubulin (Tekle et al. 2007), those in the Dakuidae are composed of actin (Brüggemann 1985b; Hooge and Rocha 2006), and the stylet of *Paratomella rubra* is composed of neither one of those molecules (own unpublished observation). Consequently, following Remane’s second homology criterion (similarity in substructure of character), the stylet as such is a homoplasious character. Notably, Xiang and Thomas (2008) showed that reconstruction signals of homolog characters are robust with regard to the analysis method used, whereas those of homoplasious characters are highly dependent on the method used, and not surprisingly, the parsimony reconstruction of the stylet is not consistent with the Bayesian reconstruction. This incongruity further applies to the pharynx. Todt (2009), who was aware of the “basal” phylogenetic position of pharynx-bearing acoels (see her Fig. 10), was unable to find any clear signs or remnants of common ancestry (other than the pharynges of Hofsteniidae and the Solenofilomorphidae). She did not provide an analysis of the characters that she thought indicative of an independent origin of pharynges; however, the same applies to Jondelius et al. (2011), who only used the presence/absence of interconnecting cells to code the diversity of the pharynges, ignoring the known variation in pharynx tube muscle layers and associated tissues, as well as in receptors. To sum up with an example that might be more current to the reader: we think that assessing the homology of eyes in the Bilateria by taking a sequence data set and running an ancestral state reconstruction by coding the eyes as present/absent, not taking the diversity of morphology into account, does not fully represent the complexity of the challenge.

Fortunately, there are robust characters by which the inner phylogeny of the Acoela can be retraced unequivocally, and these include characters of the body-wall musculature, the female copulatory organ (bursa and bursal nozzle) and sperm (Fig. 7). Sperm with cortical microtubules are found in “basal“ families; the most divergent families have, instead, axial microtubules; interestingly, taxa that are phylogenetically positioned in between these two groups have an intermediate pattern of cytoplasmic microtubules, revealing an evolutionary transformation series (Petrov et al. 2004). Within the clade possessing axial microtubules, three groups can be distinguished on the basis of the pattern of microtubules in the axonemes: the Dakuidae have two singlet microtubules in the center of the axoneme, as is typical of most cilia (9 + 2); the Mecynostomidae have, instead, only a single microtubule in this central position (9 + 1); the Convolutidae typically lack central microtubules (9 + 0) altogether (Hendelberg 1977; Raikova et al. 2001; see Fig. 4b). Achatz et al. (2010) suggest that changes in the number and position of cytoplasmic microtubules are adaptations of the sperm to accommodate passage through a bursal nozzle. Nozzles are likely bottlenecks for the sperm on their way to fertilize ova and, therefore, should lead to sperm competition. Consequently, sperm and copulatory organs, especially the bursal nozzles, are shaped according to the antagonistic co-evolution between female and male function (sexual conflict), a situation also found in other microturbellarians of the rhabditophoran genus *Macrostomum* (Schärer et al. 2011). It appears that the central microtubules of the axonemes are also subject to this pressure and have become reduced in the Mecynostomidae and Convolutidae, probably to allow bending of the sperm in more than one plane.

## Relationships with Nemertodermatida and Xenoturbellida

**Fig. 8:**
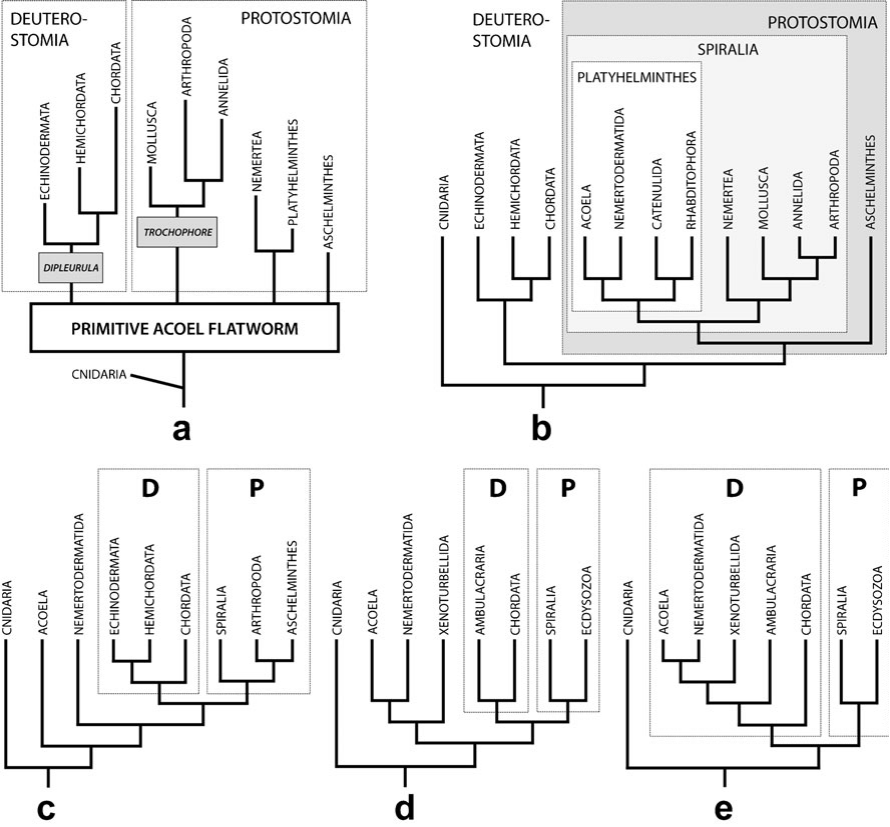
The Acoela/Acoelomorpha in different schemes of eumetazoan relationships. **a** Precladistic version assuming a small planula-like worm as the ancestor of all bilaterians and with acoels as its direct descendants (after Hyman 1951). **b** Scheme based on morphological characters; the Acoela is part of the Acoelomorpha, which is placed within the Platyhelminthes (after Westheide and Rieger 2007). **c** Phylogeny according to rDNA (Wallberg et al. 2007); the Acoelomorpha forms a paraphylum at the base of the Bilateria. **d** Phylogeny according to phylogenomics I (Hejnol et al. 2009); Acoelomorpha together with the Xenoturbellida forming a clade that is a sister group to all other Bilateria. **e** Phylogeny according to phylogenomics II (Philippe et al. 2011); the Acoelomorpha is placed within the Deuterostomia and derived by progenesis from a coelomate ancestor. Abbreviations: *d* deuterostomia; *p* protostomia

The first precladistic ideas placing acoels in the tree of life and interpreting their nature can be subsumed to the concept of the “acoeloid-planuloid hyothesis,” which was proposed by Graff (1904) and elaborated upon by Hyman (1951). This hypothesis proposed that a cnidarian-planula-like ancestor would have given rise to an acoel-like stem bilaterian that acquired bilaterality either through decompression of the body followed by a shift of the mouth from terminal to ventral (Graff) or through flattening along the oral-aboral axis and displacement of the nervous center toward one end, which became the new anterior end (Hyman). In this scenario, acoels are viewed as direct descendants of such a simple Urbilateria (Fig. 8a).

Subsequently, the theory and methodology of phylogenetic systematics (Hennig 1950, 1965) were established, and the archicoelomate theory, which postulated an ancestor with features of coelomate bilaterians (Remane 1963; Jägersten 1972), became widely accepted in Europe, whereas most US authors followed Hyman. Consequently, acoels were regarded as secondarily reduced and were classified within the Platyhelminthes, together with the Nemertodermatida forming the Acoelomorpha (Fig. 8b).

Nemertodermatids resemble acoels in general body form and the possession of a statocyst, but the statocyst bears two statoliths as opposed to the one in acoels (Ehlers 1985; Sterrer 1998). They live in mud or the interstices of sand, or are commensal (*Meara stichopus* lives in the foregut of a sea cucumber). Like acoels, they lack excretory organs and special ducts for the germ line. Despite these and other similarities between acoels and nemertodermatids, only two solid characters unite them: the ciliary rootlet system (Tyler and Rieger 1977) and the horizontal orientation of the second, asymmetric cleavage plane (Jondelius et al. 2004).

Nevertheless, knowledge of nemertodermatids is crucial in interpreting various characteristics of acoels. Extracellular matrix (ECM) is present in abundance in virtually all metazoans, but is missing under the epithelia (as basal lamina) in acoels and is also relatively scant to varying degrees in nemertodermatids (Smith and Tyler 1985a). An explanation may be that acoels substitute the mechanical properties of the basal lamina with the network of rootlets and a terminal web, which are both well developed in nemertodermatids as well (Rieger et al. 1991).

The nervous system of “basal” nemertodermatids (see Fig. 2 in Wallberg et al. 2007) consists of ring-like connectives, longitudinal neurite bundles, and a basiepithelial plexus, all positioned in the epidermis (Riser 1987; Raikova et al. 2000a, 2004b); as the nervous systems of many “basal” bilaterians are such “skin brains” (Holland 2003; see box 1), acoels likely have a more derived condition in that the connectives and neurite bundles are sunk below the body wall. The ring-shaped brain in “basal” nemertodermatids and “basal” acoels may represent the ground pattern in acoelomorphs even if most have paired ganglia complete with neuropile and rind as in other bilaterians.

As in the epithelia, ECM is missing in the parenchyma in acoels and is also relatively scant to varying degrees in nemertodermatids (Rieger et al. 1991). Considering that fixed parenchymal cells and chordoid cells are present in acoels but absent in nemertodermatids (Rieger et al. 1991) and that the differences of true parenchymal cells found between various acoelomate taxa suggests convergent evolution of such (see Rieger 1985), again, the character state found in nemertodermatids should be considered the plesiomorphic state for acoelomorphs.

In addition, the syncytial digestive system of acoels may be an extreme of conditions seen in nemertodermatids, which, while having a true epithelium and gland cells in their gut, have a small, relatively occluded lumen (Karling 1974; Smith and Tyler 1985a; see Fig. 7). A remnant of a gut lumen is evident in the acoel *Paratomella rubra* (Smith and Tyler 1985a), and various acoel species only temporarily develop a digestive syncytium after ingestion (Smith 1981).

The specialized form of the sperm in acoels (with two flagella whose axonemes are incorporated into the sperm cell) may be an adaptation to internal fertilization (Fig. 7); the sperm of the Nemertodermatida are moderately modified, presumably also for internal fertilization, but are monoflagellate like most metazoan sperm (Tyler and Rieger 1974, 1977; Hendelberg 1977; Fig. 7).

The embryonic cleavage pattern in Nemertodermatida bears resemblance to patterns in acoels, spiralian phyla (annelids, molluscs), and deuterostomes. Like that of acoels, cleavage in *Nemertoderma westbladi* takes place in a duet pattern, but starts out radial (like the cleavage patterns of deuterostomes); the micromeres later shift clockwise to produce a spiral-like pattern (Jondelius et al. 2004). Whether these differences signify an intermediate position of nemertodermatids between acoels and other animals (either spiralian or radially cleaving phyla) remains to be seen.

All of these features point to the Acoela being rather derived in comparison to the Nemertodermatida, which seem to have retained more characters in states more like those of other basal bilaterians (Tyler and Rieger 1977; Smith and Tyler 1985a; Tyler 2001). Some of these differences—for example, the digestive syncytium, the possession of a pharynx, or the position of the central nervous system below the body wall—may have facilitated diversification in ways not available to nemertodermatids. That diversification is now reflected in the approximately 400 described species compared to only 8 in the Nemertodermatida.

Even though a separate placement of the Acoelomorpha from the Platyhelminthes has been suggested based on morphological characters (Smith et al. 1986) and cladistic analyses of such (Haszprunar 1996), it was the comparison of sequence data on rDNA of the acoel *Paratomella rubra* (Ruiz-Trillo et al. 1999) and some other acoel species with that of other metazoan phyla that paved the way for the acceptance of such a split and the position of acoels at the very base of the Bilateria (Carranza et al. 1997; Ruiz-Trillo et al. 1999; Jondelius et al. 2002; Telford et al. 2003; Wallberg et al. 2007; Jondelius et al. 2011; Fig. 8c). Surprisingly, in these analyses acoels and nemertodermatids were split (Fig. 8c); however, data from amino acid sequences of mitochondrial genomes (Ruiz-Trillo et al. 2004; Mwinyi et al. 2010) and ESTs (Hejnol et al. 2009; Philippe et al. 2011) did re-establish the high probability of a sister group relationship between Acoela and Nemertodermatida and the validity of the Acoelomorpha (Figs. 8d, e). To place the Acoelomorpha, rDNA genes seem unsuitable because of their high A + T content and rather truncated and modified nature (Mallatt et al. 2010). Additionally, even though base composition bias or long branch attraction could be excluded to affect the placement of the Acoela in Wallberg et al. (2007), the limited number of genes likely makes us follow the evolution of these genes more than the organisms from which they have been sequenced.

Unfortunately, the content and order of mitochondrial genomes are unsuitable to infer the phylogenetic position of acoelomorphs because the existing data are either too scarce or, in the case of the complete mitochondrial genome of the highly derived acoel *Symsagittifera roscoffensis*, too divergent (Mwinyi et al. 2010). Consequently, the most reliable hypotheses based on molecular data come from analyses of amino acid sequences in either mitochondrial genomes or EST collections, and these suggest that the Acoelomorpha is either (1) the earliest offshoot of the Bilateria (Hejnol et al. 2009, Mwinyi et al. 2010) or (2) in a sister group relationship with *Xenoturbella bocki* as the earliest offshoot of the Bilateria (Hejnol et al. 2009; Fig. 8d); or 3) together with *Xenoturbella bocki* among the deuterostomes (Philippe et al. 2011; Fig. 8e).

But what is *Xenoturbella bocki*? It constitutes, together with *Xenoturbella westbladi* (Israelsson 1999), the enigmatic Xenoturbellida (Bourlat et al. 2006) and is a remarkably simple worm, lacking organs other than an anterior statocyst. It is found on deep marine muds off the coasts of Scandinavia and Scotland. While considerably larger than acoels (measuring up to 4 cm in length), it has been linked to them through its simple morphology (worm shape, acoelomate structure, single opening to the gut), similarity in its nervous system, and lack of excretory organs and tissue enclosing the germ cells (Westblad 1949; Hyman 1959). More similarities are discernible through electron microscopy, especially in the shape of the cilia, their axonemal termination patterns, and their rootlets (Pedersen and Pedersen 1986, 1988; Franzén and Afzelius 1987; Lundin 1998). *Xenoturbella* has pulsatile bodies (degenerating epidermal cells) that appear much like those of acoelomorphs (Lundin and Hendelberg 1996; Lundin 2001); the cellular but unciliated nature of its gut is reminiscent of nemertodermatids, and its lack of a somatogastric nervous system (Raikova et al. 2000b) is similarly reminiscent. *Xenoturbella* also shows even stronger affinity with hemichordates and echinoderms through molecular sequence similarity (Bourlat et al. 2003, 2006, 2009) and morphological similarity of its epidermis and statocyst (Reisinger 1960; Pedersen and Pedersen 1986; Stach et al. 2005). However, the occurrence of monociliated parietal cells in the statocysts of apodous sea cucumbers and *Xenoturbella* most likely originated independently (Ehlers 1997).

Interestingly, Philippe et al. (2011) linked *Xenoturbella* to both Acoelomorpha and Ambulacraria (i.e., echinoderms + hemichordates) with sequence data of amino acids in a genomic set and mitochondrial genes. Further support for a close relationship among acoelomorphs, *Xenoturbella*, and deuterostomes comes from shared specific microRNAs, a shared sperm protein (Philippe et al. 2011), and a shared GNE kinase (De Mendoza and Ruiz-Trillo 2011), all of which are present only in these groups. As a cautionary note, however, we stress that the nature of microRNAs is rather problematic inasmuch as losses constantly occur and in the groups in question the data have not been backed up by a genome; the RSB66 sperm protein and epimerase could also have been lost specifically in the protostomes (De Mendoza and Ruiz-Trillo 2011). Additionally, the bootstrap supports for the Xenacoelomorpha are low in the analyses of Hejnol et al. (2009) as well as in Philippe et al. (2011), and one needs to explain the difference in mitochondrial gene order and different codons for serine in the acoelomorphs in comparison to deuterostomes (Bourlat et al. 2003). Furthermore, the morphological features that are similar between acoelomorphs and xenoturbellids could all be shared plesiomorphies or convergent adaptations to the benthic life of small worms (see Pardos 1988 for ciliary tips and rootlets). Taking all these facts into consideration, we are not opposed to but reluctant to accept the validity of a clade Xenacoelomorpha.

## What is primitive in Acoelomorpha?

Whether the ancestor to all living bilaterians was a simple acoelomate worm or a more complex coelomate is a long-standing and ongoing debate (Rieger 1986; Holland 2003; De Robertis 2008). Proponents of the former hypothesis commonly refer to the “acoeloid-planuloid hyothesis” (e.g., Salvini-Plawen 1978; Baguñà and Riutort 2004; Wallberg et al. 2007; Hejnol and Martindale 2008a) and interpret acoelomorphs as “conserved” descendants of a simple urbilaterian and the basic acoelomorph body plan—simple basiepidermal nervous system and lack of anus, lining tissue over germ cells, and excretory organs as well as direct development—as primitive in the line leading to the rest of the Bilateria. Proponents of the archicoelomate theory (complex coelomate ancestor) usually suggest that acoelomorphs have acquired their recent organization through secondary loss of many features. The recently recovered position as sister group to the Ambulacraria within the Deuterostomia would support this idea because it is easier to loose characters such as through-gut, nephridia, deuterostomy, and gill slits once opposed to evolve them independently twice within the Deuterostomia. Comparable scenarios have been shown to occur in protostomes through either reduction of the coeloms or progenesis in a coelomate animal with acoelomate or pseudocoelomate larvae or juveniles (Rieger 1980, 1986; Schuchert and Rieger 1990; Fransen 1980a, b; Tyler 2001). One might oppose the latter proposition that the larvae of deuterostomes are coelomate and that the assumption of progenesis does not work in this case. However, the key point is that in acorn worms, pterobranchs, and echinoderms, mesoderm and coelomic cavities do not just appear through enterocoely from the archenteron but also through schizocoely and delamination (Peterson et al. 1999; Ruppert et al. 2004). Consequently, by suppressing the mesenchymal-epithelial transition or forestalling maturity to a developmental stage earlier than the mesenchymal-epithelial transition, the acoelomate condition could also be accomplished in a “deuterostome-like” coelomate.

Unfortunately, no morphological feature helps us to unequivocally decide between the two scenarios outlined above and the same applies to results from Evo-Devo studies.

The central nervous system with basiepidermal ring commissures and major neurite bundles could just as easily reflect features of the urbilaterian as descent from a basal deuterostome. The homology of its subunits with structures of other bilaterians remains a matter of debate (Rieger et al. 1991; Raikova et al. 1998, 2000a; Bery et al. 2010). Semmler et al. (2010) found *SoxB1* to be widely expressed in the developing brain of *S. roscoffensis*, a finding that is consistent with its expression in developing neural structures throughout cnidarians and bilaterians. However, *SoxB1* is not, strictly speaking, a “brain marker” in that it is also expressed in the apical organ of the larvae of an acorn worm (Taguchi et al. 2002). Finally, the anterior-to-posterior development of the nervous system of acoels and its similarity with the oral-aboral gradient of the nervous system of cnidarians has led some to speculate that it reflects the first steps in centralization of the nervous systems of the Bilateria (Marlow et al. 2009; Semmler et al. 2010).

The proposed ancestral role of the ParaHox genes is the anteroposterior patterning of the digestive system; in particular, *cdx* shows conserved expression in a posterior ectodermal domain that is associated with the formation of the hindgut, and this was taken to mean that the anus of all Bilateria was homologous (Hejnol and Martindale 2008b). In the acoel *C. longifissura*, which like all acoels lacks an anus, *cdx*, together with other homologs of bilaterian hindgut markers such as *brachyury* (*bra*), *orthopedia* (*otp*), and the homeobox gene *nk2.1*, is expressed in a posterior ectodermal domain of juveniles in tissue that later forms the male gonopore (Hejnol and Martindale 2008b).

These findings have profound implications for the evolution of a through-gut. While the expression of genes such as *goosecoid* and *brachyury* in the mouth region of not only acoels and the rest of the Bilateria but also cnidarians indicates homology of the anterior gut opening throughout the Metazoa, the presence of hindgut genes in the region of the future male gonopore in acoels may be interpreted as showing independent, multiple origins of the anus in the bilaterians or of secondary reduction of the hindgut in acoels and its cooption for the gonopore (cf. Gnathostomulida, which have secondarily lost the anus—Knauss 1979). Hejnol and Martindale (2008b) followed Reisinger (1961) in suggesting that the anus evolved as a common opening of the gut and gonoducts (cloaca). If, however, these genes have more general morphogenic functions (if, for instance, *brachyury* simply organizes infolding of epithelia), then these speculations may be premature.

Asaccate gonads can be interpreted as a primitive character of the Acoelomorpha. However, this feature is also found in catenulid platyhelmiths (Rieger et al. 1991) and in the ovaries of several subgroups of Gnathifera, namely the Gnathostomulida (Mainitz 1983) and the Micrognathozoa (Kristensen and Funch 2000). Noteworthily, stromal cells can be found in gonads of the “basal” acoels *Diopisthoporus* ssp. (Westblad 1945, 1948; Smith and Tyler 1985a) and *Nemertoderma* sp. (Tyler and Rieger 1977), perhaps being vestiges of a more primitive condition.

Acoelomorphs appear to fundamentally lack excretory organs, and this is routinely taken to be a primitive feature (Jondelius et al. 2002). If acoelomorphs are progenetic or reduced descendants of a coelomate ancestor that would have relied on a coelomic cavity to produce primary urine, then loss of the cavity in progenesis would have left acoelomorphs without any obvious excretory organ. Deuterostomes do not have protonephridia, and their absence from acoelomorphs could be taken as further evidence in favor of their proper placement outside the protostomes, as the basal-most Bilateria or in the Deuterostomia.

Surveys of the homeodomain via degenerate PCR have identified three bona fide Hox genes in acoels—one anterior, one central, and one posterior—and only the homolog of the posterior ParaHox gene *caudal* (*cdx*—Hejnol and Martindale 2009; Moreno et al. 2009; for discussion see above). As in all Bilateria, the acoel Hox genes are expressed in staggered spatial domains along the anteroposterior axis; however, they are all expressed at approximately the same developmental stage, i.e., after gastrulation during embryonic development and at bud initiation during asexual reproduction (in this latter case with the exception of the central Hox gene, the expression of which is slightly delayed with respect to the anterior and posterior Hox genes). The lack of temporal colinearity in Hox gene expression is best explained by the lack (or disruption) of the Hox gene cluster in the Acoela (Moreno et al. 2009).

The anterior and central Hox genes are expressed in the neuroectoderm of the developing embryo of *Convolutriloba longifissura,* and in the cerebral ganglion and developing neurite bundles of the related species *Convolutriloba retrogemma* and *Symsagittifera roscoffensis* (Hejnol and Martindale 2009; Moreno et al. 2009; Sikes and Bely 2010). Evidence of the neural patterning nature of the anterior and central Hox is reinforced by the overlapping expression of the neural gene *SoxB1* in *C. longifissura* and *S. roscoffensis* (Hejnol et al. 2009; Semmler et al. 2010; our personal observations). The posterior Hox gene is expressed in the three germ layers in *C. longifissura* and in the posterior peripheral parenchyma in *S. roscoffensis* and *I. pulchra*. Its function has been tested in the latter species by RNA interferrence, during adult homeostasis, regeneration, and juvenile development. The gene is necessary for egg maturation and the correct development and maintenance of the posterior musculature, while its function is less clear in the posterior nervous system (Moreno et al. 2010).

Though the most parsimonious interpretation of the data is that acoels bear the primitively minimal set of Hox genes and are themselves a basal clade within the Bilateria, it is also possible that the low number of Hox genes is concordant with a secondary simplification of the body plan. The fact that the left complement includes one Hox gene of each class (anterior, central, and posterior) could be attributed to a reduction that leaves only a minimal set compatible with bilateral organization (Moreno et al. 2011).

The paucity of microRNAs in *S. roscoffensis* and *Childia* and especially the lack of key microRNAs necessary for organogenesis such as *miR-1* (heart) or *miR-9* (brain) correlate with a basal position of acoels and support the aceloid-planuloid hypothesis (Sempere et al. 2006, 2007). However, Philippe et al. (2011) found four additional microRNAs in the more basal acoel *Hofstenia miamia* and thus showed intraphylum variability and that microRNAs may have been lost in most acoels.

## Conclusion

Certainly the Acoelomorpha does not belong in the Platyhelminthes, and Acoela + Nemertodermatida is a monophylum. If it were a paraphylum at the base of the Bilateria as suggested by some studies either their similarities in development, ciliary structure, and rootlet system must have originated independently twice, which is very unlikely, or these traits would have to be plesiomorphic for bilaterians, which is even more unlikely. The Acoelomorpha are, furthermore, not members of the protostomes, as they have never been recovered within this clade in molecular sequence analyses; the absence of protonephridia and the endomesodermal origin of muscles further corroborate this assumption.

To us it is clear that the ancestor common to acoelomorphs and other bilaterians was quite different from a present-day acoel or nemertodermatid. In analyses of ribosomal genes and phylogenomic approaches, acoels and nemertodermatids have very long branches (see figure 2 in Wallberg et al. 2007 and figure 3 in Philippe et al. 2011), and while a long branch does not necessarily mean a variation in complexity, it by definition means that the molecules analyzed are quite different from the inferred ancestral state. As an organism and its molecules evolve as an entity, it is difficult to comprehend how an organism could evolve slowly while its molecules are evolving fast. Not a single so-called “living fossil” has shown an extraordinary branch length yet in any analysis (e.g., Webster et al. 2006 for priapulids), and animals that are quite different from the inferred ancestral state show relatively long branches compared to the former (e.g., Struck et al. 2011 for myzostomids).

Animals with a branch length comparable to those of the Acoelomorpha analyzed under the same conditions by Philippe et al. (2011) are suggestively “simple”—platyhelminths, rotifers, and cycliophorans, all believed to be small and simplified by regressive evolution. The descent of the Acoelomorpha from a more “complex” or better quite different ancestor would elegantly account for the long branches in all molecular investigations and their peculiar morphology.

Whether one accepts Acoelomorpha as the sister group to the remaining Bilateria or prefers their placement in the Deuterostomia, together with the placement of the Chaetognatha either basal to ecdysozoans and spiralians (Marlétaz et al. 2008) or nested within one of those clades (see Harzsch and Wanninger 2010 for review), it throws the value of the terms “Deuterostomia” and “Protostomia” into question. Reflecting on the nervous system and development of the Acoelomorpha and Chaetognatha, it might well be anticipated that the term “Protostomia” should be replaced with the term “Gastroneuralia” (Schimkewitsch 1891; Ulrich 1951) and that a new term should be introduced for the clade comprising Ambulacraria and Chordata (and probably Xenacoelomorpha).

## Future perspectives

We need more information before the Acoelomorpha can be placed definitely in bilaterian phylogenies and before we can reconstruct the appearance of the ancestor common to the Acoelomorpha and other bilaterians. Information now available from EST collections of acoels (*C. longifissura*, *I. pulchra*, *N. fusca*, *S. roscoffensis*), nemertodermatids (*Meara stichopi*, *N. westbladi*), and *Xenoturbella bocki*, as well as from microRNA libraries (*Hofstenia miamia*, *N. fusca*, *S. roscoffensis*) and BAC (genomic) libraries (*S. roscoffensis*), has yet to be fully tapped. Whole-genome projects on various acoelomorphs and *X. bocki* are pending. Among newer techniques from which we can expect novel phylogenetically relevant information are gene knock-down protocols with double-stranded RNA (as has been applied to *I. pulchra*: De Mulder et al. 2009; Moreno et al. 2010 and *H. miamia* (personal communication Mansri Srivastava)), cryoelectron microscopy (Salvenmoser et al. 2010), immunocytochemistry, staining for mitotic cells (Gschwentner et al. 2001; De Mulder et al. 2009), and in situ hybridization. For *in situ* probes a significant “breakthrough” has been made that provides access to the embryo through the eggshell (Hejnol and Martindale 2008a; Hrouda 2007; De Mulder 2009). However, a method with which the embryo can be made accessible for double-stranded RNA without damaging or alternating the development of the embryo is still required.

The production of transgenic animals would also be a significant development. The creation of stable transgenic lines would allow us to link gene expression and function to morphogenetic events underlying the development of defined structures. A major challenge in transgenesis is the production of germ line-transgenic specimens able to transmit the transgene to the offspring, avoiding the problems associated with mosaicism. The availability of technologies for functional analysis in these worms is essential to decipher whole gene regulatory networks (GRN) and infer putative ancestral regulatory states controlling cell type and tissue differentiation as well as the developmental origins of defined body plan features (Davidson 2011).

The number of immunocytochemical markers specific to acoelomorphs remains relatively limited—the production of a library of monoclonal antibodies, as has been achieved for other flatworms (Bueno et al. 1997; Ladurner et al. 2005), from carefully selected species would be indispensable. In addition, having a good embryo microinjection technology would help when it comes to lineage tracing and knock-down in specific lineages; 4D microscopy would be beneficial in analyzing such lineages.

All the tools mentioned above need to be applied with an eye to testing the proposed positions of the Acoelomorpha and evaluating the apomorphic or plesiomorphic state of morphological and molecular characters under investigation. Pinpointing this is critical to understanding one of the most important stages in animal evolution. However, regardless of their precise phylogenetic position, they are highly valuable for comparative analyses of genomes and gene features, to unravel how genome and morphology are linked, and as a source of comparison to understand bilaterian features such as the multiciliated epidermis, acoelomate body plan, spiral cleavage, the “centralization” of the nervous system, and its immersion below the body wall.
